# Prospectus of Massie’s Eclectic Southern Practice

**Published:** 1853-04

**Authors:** 


*Prospectus of Massie's Eclectic Southern Practice.*—We are gratified to perceive, from the "Prospectus" before us, that we are about to be favored with a work on the Diseases of the South, by a gentleman every way competent for the undertaking. Dr. Massie, of Texas, has been solicited by a large number of his brother practitioners, who are well acquainted with his high qualifications, to write a work on the Theory and Practice of Medicine—a work "presenting the various modifications which diseases assume in Texas" (and the South generally.—*Ed.*) The material for such a work is varied, abundant, and needs but some skillful hand to gather the rich harvest which is now ripe, to digest and arrange it for our common profession. As yet little has been done, save through the pages of a few medical periodicals, to advance, in the estimation of our cotemporaries, the science of Medicine in the South. For works, whether in Surgery or the Practice of Medicine, we have heretofore looked to friends of the North and of Europe; when we should begin to put forth our own efforts, and build up a system of practice adapted to our climate, the peculiar nature of our diseases, and the wants of the profession. Facts in abundance are at hand, as rich as they are diversified—diseases unknown to other latitudes and other climes daily fall under our observation, and demand our care, yet we have but few records, and no standard work, to which we can turn for instruction and advice, treating of our peculiar diseases, in times of doubt and difficulty. To possess a work embodying the history, the peculiar features of our epidemic and endemic diseases, and also their pathology and therapeutics, is indeed a desideratum which has long been felt by the profession throughout the South. We are rejoiced, therefore, to learn that a physician—a man of, and belonging to us—one of extensive experience, enlarged and liberal views—of much reading and reflection—a scholar, in short, is about to undertake to supply this desideratum to the profession.

The work will be brought out in the course of twelve months, at least, the price of which will be fixed at five dollars per copy.

We look forward to the appearance of Dr. Massie's new work with unusual interest, and shall hail its publication as the establishment of a new epoch in Southern Medicine.—*N. Orleans Med. Journal*, *(Editorial.)*

